# The Impact of Automatic Exposure Control Technology on the In Vivo Radiation Dose in Digital Mammography: A Comparison Between Different Systems and Target/Filter Combinations

**DOI:** 10.3390/diagnostics15101185

**Published:** 2025-05-08

**Authors:** Ahmad A. Alhulail, Salman M. Albeshan, Mohammed S. Alshuhri, Essam M. Alkhybari, Mansour A. Almanaa, Haitham Alahmad, Khaled Alenazi, Abdulaziz S. Alshabibi, Mohammed Alsufayan, Saleh A. Alsulaiman, Maha M. Almuqbil, Mahmoud M. Elsharkawi, Sultan Alghamdi

**Affiliations:** 1 Department of Radiology and Medical Imaging, Prince Sattam bin Abdulaziz University, Al-Kharj 16278, Saudi Arabia; m.alshuhri@psau.edu.sa (M.S.A.); e.alkhybari@psau.edu.sa (E.M.A.); 2 Department of Radiological Sciences, College of Applied Medical Sciences, King Saud University, Riyadh 11451, Saudi Arabia; salbeshan@ksu.edu.sa (S.M.A.); malmanaa@ksu.edu.sa (M.A.A.); hnalahmad@ksu.edu.sa (H.A.); kenazi@ksu.edu.sa (K.A.); abdalshabibi@ksu.edu.sa (A.S.A.); 3 Radiology Department, Altakassusi Alliance Medical LLC, King Fahad Medical City, Riyadh 12231, Saudi Arabia; mohammed.alsufayan@aaml.com.sa; 4 Radiology & Medical Imaging Department, Imam Abdulrahman Alfisal Hospital, Cluster One, Riyadh 14723, Saudi Arabia; salsulaiman7@gmail.com; 5 Cancer Control Program, Ministry of Health, Riyadh 12628, Saudi Arabia; dr.maham.mm@gmail.com; 6 Department of Health Programs, Ministry of Health, Riyadh 12628, Saudi Arabia; m10001000c@gmail.com; 7 Radiology and Nuclear Medicine Department, Security Forces Hospital, Riyadh 11481, Saudi Arabia; sultan-661@hotmail.com

**Keywords:** digital mammography, mammography technology, mean glandular dose, target/filter combination, AEC optimization, compressed breast thickness

## Abstract

**Background/Objectives**: Digital mammography is widely used for breast cancer screening; however, variations in system design and automatic exposure control (AEC) strategies can lead to significant differences in radiation dose, potentially affecting the diagnostic quality and patient safety. In this study, we aimed to determine the effect of various mammographic technologies on the in vivo mean glandular doses (MGDs) that are received in clinical settings. **Methods**: The MGDs and applied acquisition parameters from 194,608 mammograms, acquired employing AEC using different digital mammography systems (GE, Siemens, and two different models of Hologic), were retrospectively collected. The potential variation in MGD resulting from different technologies (system and target/filter combination) was assessed employing the Kruskal–Wallis test, followed by Dunn’s post hoc. The AEC optimization of acquisition parameters (kVp, mAs) within each system was investigated through a multi-regression analysis as a function of the compressed breast thickness (CBT). The trend line of these parameters in addition to the MGD and source-to-breast distance were also plotted and compared. **Results**: There were significant variations in delivered doses per CBT based on which technology was used (*p* < 0.001). The regression analyses revealed system-specific differences in AEC adjustments of mAs and kVp in response to CBT changes. As the CBT increases, the MGD increases with different degrees, rates, and patterns across systems due to differences in AEC strategies. **Conclusions**: The MGD is affected by the applied technology, which is different between systems. Clinicians need to be aware of these variations and how they affect the MGD. Additionally, manufacturers may need to consider standardizing the implemented technology effects on the MGDs.

## 1. Introduction

Mammography is the primary evidence-based method for detecting breast cancer before the appearance of any signs or symptoms [1]. The mammography screening for women over 40 can reduce breast cancer mortality rates by 15–40% [2,3]. However, while effective, mammography involves the use of X-ray radiation, which is ionizing and carries potential risks [4]. The glandular tissue in the breast, comprising acinar and ductal epithelium along with associated stroma, is particularly radiosensitive [5]. Consequently, optimizing the delivered breast dose is crucial in controlling the potential harm to glandular tissue from mammography examinations. 

The mean glandular dose (MGD) is a key metric in breast imaging, representing the average dose that is absorbed in the central glandular region of the breast. International radiation protection bodies, such as the International Commission on Radiological Protection and the International Atomic Energy Agency, recommend using MGD as the primary indicator of radiation risk in mammography [6,7].

In mammography, different variables can influence the MGD. The exposure current (mAs) is positively related to the dose [8]. Additionally, a larger compressed breast thickness (CBT) usually results in a higher MGD [8,9]. Moreover, mammographers apply a compression force (CF) to spread the breast tissues and reduce the breast thickness, which helps in improving image quality and is found to reduce the radiation dose [8]. Nevertheless, the X-ray beam energy can affect the MGD as well; a higher energy allows for further penetration and may influence the MGD [10]. The machine might need to produce an X-ray beam with higher energy with a denser or larger CBT to increase its penetration. The X-ray beam energy spectrum is determined by the kVp and target/filter combination. To optimize the dose and image quality, the energy spectrum can be adjusted automatically by the machine using the automatic exposure control (AEC) feature, which adjusts the kVp and target/filter combination [11]. The fact that each manufacturer provides different target/filter materials can cause variation between delivered MGDs based on which manufacturer’s system was used. Moreover, the technology that is implemented by manufacturers to set the acquisition parameter combination during the AEC may vary and result in different doses. 

With the transition from screen film to digital mammography, which allows for the adjustment of the display contrast of the acquired digital images, the use of higher X-ray energies has gained more interest, as it helps in optimizing the dose and reducing both noise and the detector’s dynamic range [10,11,12,13,14]. Accordingly, manufacturers started to employ more material combinations of anodes (targets) and filters that can provide higher energies. Thus, understanding the influence of acquisition parameters on the MGD under each target/filter combination is needed. 

Understanding the clinical implications of dose variability is crucial for optimizing patient care. Even small differences in the MGD can influence long-term risks, such as the potential for radiation-induced malignancies, particularly in populations undergoing frequent screenings. Conversely, insufficient exposure may compromise the image quality and hinder early cancer detection. Therefore, accurately quantifying and minimizing dose variability not only addresses technical optimization challenges but also enhances patient safety and the diagnostic efficacy. This underscores the need for a detailed evaluation of how different systems and target/filter combinations impact the MGD in real clinical settings.

As mentioned earlier, the optimization of the radiation dose in digital mammography depends on various factors, including the system type, target/filter combination, and CBT. The CBT represents the main variable among women that the system can measure, and thus, it influences the selection of imaging parameters, such as kVp, mAs, and target/filter material, which in turn affect the MGD. Therefore, studying these parameters in relation to the CBT would be insightful.

Although useful information has been gained from phantom studies, in vivo studies are still important, as human bodies and clinical situations are more complicated. Additionally, while some acquisition parameters in mammography have been studied previously, the impact of some parameters, such as the source-to-breast surface distance (SBD) and focal spot, has not been investigated enough, especially in vivo. 

Having many factors and technologies influencing the MGD complicates the understanding of how these factors impact the dose and how to optimize it. Thus, in this work, the aim was to clarify their effects on the MGD that is delivered to imaged women within clinical settings through studying a set of different commonly used mammogram technologies and acquisition parameters.

## 2. Materials and Methods

### 2.1. Data Collection

Ethical approval was granted before starting the project. As summarized in Table 1, data including 194,608 mammogram images, acquired using four different machine manufacturers/models, were retrospectively retrieved. The data were collected from centers that perform regular quality control tests for their mammography machines by independent medical physicists at each site and, where applicable, supervised by a centralized medical physics office. All contributing centers follow the ACR accreditation guidelines for mammography QC. An in-house written code in MATLAB (MathWorks, Natick, MA, USA; version 9.3) was used to extract the MGD values measured by the machine, the used acquisition parameters (mAs, kVp, anode (target) and filter materials, focal spot, exposure control mode, CBT, CF, and SBD), machine information (manufacturer and model names), and filter thickness from the images’ DICOM file header. Only images that were acquired with the AEC mode were included to reduce subjective external “operator” factors and focus on how the implemented AEC technology optimizes doses. The machines used in our datasets are Hologic (two models; Selenia Dimensions and Lorad Selenia), GE (Senographe Essential), and Siemens (Mammomat Inspiration). Each of these machines employs a different combination of target/filter materials (see Table 1).

### 2.2. Assessing Dose Variation Among Mammography Machines

To illustrate the system performance variation in dose optimization for different breast sizes, a bar chart was used to show the distribution of delivered MGDs across different CBT ranges for each mammography system. 

To draw a statistical conclusion about the existence of dose variation among technologies (system and target/filter combination), the Kruskal–Wallis rank sum test was implemented, and Epsilon-squared was computed to measure the magnitude of the differences. The Kruskal–Wallis test was followed by Dunn’s post hoc test to identify specific pairwise differences, and Holm correction was applied to adjust *p*-values for multiple comparisons. To minimize the effect of variability in women’s breast thickness, the MGD values were normalized to their corresponding CBTs before the analysis, allowing for a more standardized comparison across systems. Because only some system datasets include 0.1 mm focal spot data, those 0.1 mm datasets were excluded from the normalized MGD-per-CBT analysis to avoid uneven dose comparisons due to focal spot effects. However, 0.1 mm data were retained for the subsequent AEC parameter optimization trend analyses to illustrate the ACE behavior under focal-spot-specific settings. Before analysis, outliers were removed from each dataset. These statistical analyses were performed using R (R Foundation for Statistical Computing, Vienna, Austria; version 4.3.0).

### 2.3. Analysis of AEC Optimization of Acquisition Parameters Across Mammography Systems

A multi-regression analysis was performed to evaluate the performance of AEC as a response to different women’s breast thickness. The acquisition parameters (mAs, kVp, SBD, and CF) were regressed against the corresponding CBT. The study was performed for each employed technology (machine model and their combination of anode/filter and focal spot) to assess any potential variation. To facilitate the comparison, the trendlines of all employed technologies were plotted on the same figure. Before regression, the datasets were processed to remove outliers. The regression analysis was performed using a custom MATLAB function, which tests a set of candidate models and automatically selects the one with the highest coefficient of determination (R^2^). This approach allowed us to characterize the trends of each parameter as a function of the CBT while accounting for system-specific differences. 

Further, similar regression analyses were conducted to assess the MGD values resulting from each system’s AEC response at each CBT.

## 3. Results

### 3.1. Variation in MGD Across Machines

**Figure 1:**
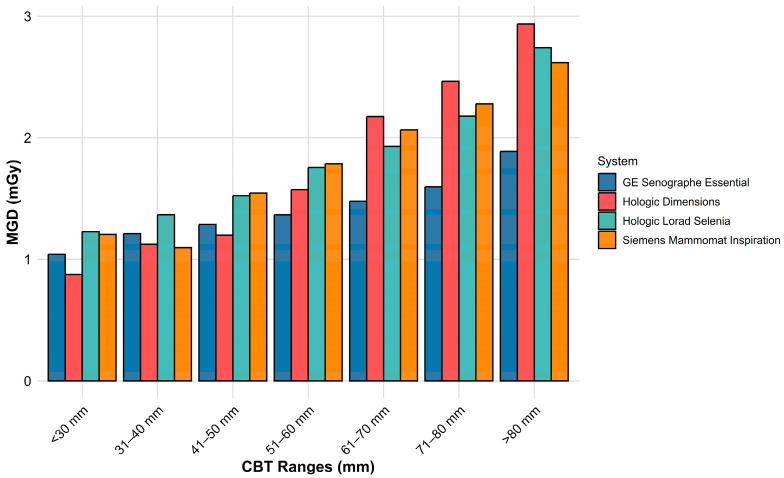
Bar chart illustrating the mean glandular dose (MGD) across different compressed breast thickness (CBT) ranges for various mammography systems, including GE Senographe Essential, Hologic Dimensions, Hologic Lorad Selenia, and Siemens Mammomat Inspiration.

Figure 1 shows the average MGD delivered to different CBT ranges by each mammography system. In general, as the CBT increases, the MGD also increases, regardless of the system used. However, the systems exhibit varying degrees of dose optimization. Hologic systems (Dimensions and Lorad Selenia) show a steeper dose increase with increasing CBTs. The Hologic Dimensions system provides the lowest MGDs at smaller CBTs before its doses increase sharply to give the highest MGDs for larger CBTs. GE Senographe Essential exhibits a more gradual increase in MGD across CBT ranges and provides the lowest MGD for large CBTs. Siemens Mammomat Inspiration displays an intermediate trend, with dose adjustments following a moderate curve.

The Kruskal–Wallis test showed significant differences (*p* < 0.001) in normalized MGD per CBT across different systems and target/filter technologies. The Epsilon-squared effect size (0.093) suggests a substantial impact of these factors on the radiation dose. 

**Figure 2:**
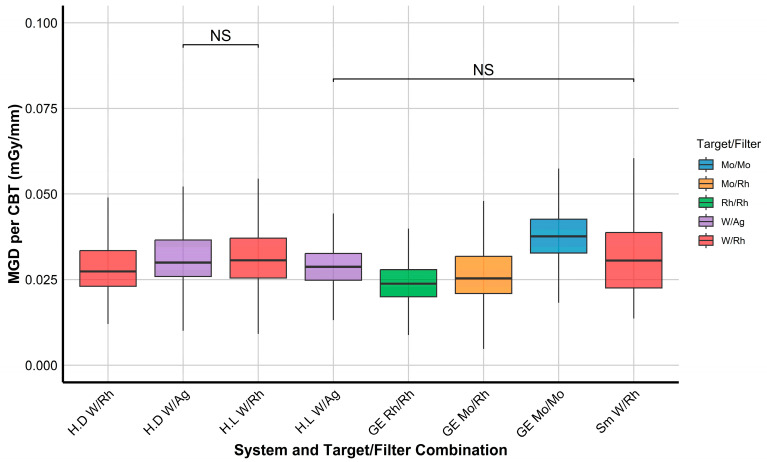
Boxplots showing the varying effects of using different mammographic technologies (target/filter combination with different machine models) on the mean glandular doses (MGDs), which were normalized to their corresponding compressed breast thickness (CBT). Non-significant (NS) comparisons are marked. Each target/filter combination is assigned a unique color to facilitate visual interpretation. Note: all data used in this figure are acquired with 0.3 focal spots, but the filter thickness varies across systems (Hologic Dimensions (H.D) and Siemens (Sm) use 0.05 mm for all filters, Hologic Lorad (H.L) uses 0.057 mm, with GE Mo/Mo (0.03 mm), Mo/Rh (0.025 mm), and Rh/Rh (0.025 mm)).

Figure 2 shows a boxplot that compares the MGD per CBT across systems and target/filter combination groups. Dunn’s post hoc test results after the Kruskal–Wallis test identified significant differences in the majority of pairwise comparisons (26 out of 28), referring to the variability in dose optimization across different systems and target/filter combinations. Non-significant differences were found only between Hologic Dimensions W/Ag (0.05 mm) and Hologic Lorad W/Rh (0.057 mm) (*p* = 0.38), and Hologic Lorad W/Ag (0.057 mm) and Siemens W/Rh (0.05 mm) (*p* = 0.98). The comparisons between Hologic Dimensions W/Ag (0.05 mm) and Siemens W/Rh (0.05 mm) (*p* = 0.003), and Hologic Dimensions W/Rh (0.05 mm) and Siemens W/Rh (0.05 mm) (*p* = 0.009) were of relatively lower significance compared to the other pairwise comparisons, which rendered highly significant differences (*p* < 0.001). 

As shown in Figure 2, the lowest and highest MGD per CBT were given by the GE system depending on which target/filter combination was used. The GE with Mo/Mo resulted in the maximum MGD per CBT, while the GE with Rh/Rh provided the lowest MGD for a given CBT, followed by the GE Mo/Rh.

### 3.2. AEC Parameter Optimization Variation Across Systems

**Figure 3:**
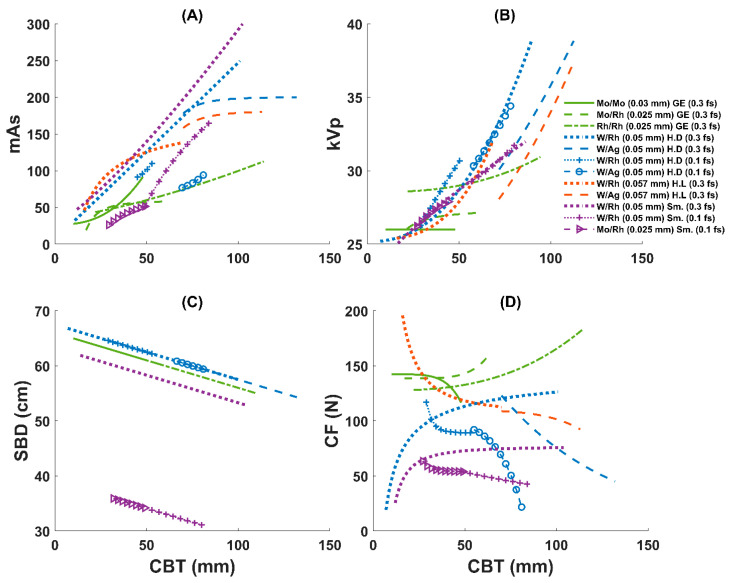
Trends in (**A**) mAs, (**B**) kVp, (**C**) source-to-breast distance (SBD), and (**D**) compression force (CF) versus compressed breast thickness (CBT) for multiple target/filter (and filter thickness) combinations across GE, Hologic Dimensions (H.D), Hologic Lorad (H.L), and Siemens (Sm.) systems. Data with different focal spots (f.s) for some systems are also plotted separately (data with markers acquired with 0.1 f.s). Colors are assigned by manufacturer (GE, green; Hologic Dimensions, blue; Hologic Lorad, orange; Siemens, purple), and line styles differentiate target/filter combinations.

Figure 3 illustrates how each manufacturer’s AEC system responds to changes in the CBT. In the figure, trends in mAs, kVp, SBD, and CF are plotted versus CBT for multiple target/filter combinations, with various filter thicknesses being used across the studied systems.

In general, mAs rises with the CBT for all systems, reflecting the additional exposure that is needed for thicker breasts. Although each manufacturer demonstrates this same overall trend, the rate of increase differs across target/filter combinations. GE’s curves typically increase in mAs at a moderate pace over narrower CBT ranges, whereas the curves of Siemens 0.3 focal spot data and Hologic Dimensions and Lorad reach higher mAs values, especially at larger thicknesses. Siemens 0.1 focal spot data tend to be used with low mAs, with a slight increase with W/Rh and a sharper increase with Mo/Rh. Hologic Dimensions with 0.1 focal spot data tend to occupy an intermediate range of mAs, although W/Ag is used with higher doses at lower CBTs compared to when W/Rh is in use. In general, 0.1 mm focal spot data often require somewhat lower mAs at a given thickness compared to 0.3 mm focal spot modes, although this pattern is not uniform across all systems. Both Hologic models use W/Ag for larger CBTs (≥70 mm), which seems to allow for lower mAs compared with W/Rh at these CBT values. However, the used mAs with W/Ag is higher in the Hologic Dimensions (which uses a 0.05 mm filter thickness) than in Hologic Dimensions Lorad (which uses a 0.057 mm filter thickness) at similar CBTs.

For kVp, it also tends to increase as the CBT grows, but with varying extents among manufacturers and target/filter modes. GE’s Mo/Mo, Mo/Rh, and Rh/Rh lines typically shift only a few kilovolts, whereas Hologic often covers a broader kVp span, indicating a more dynamic beam quality adjustment. When 0.1 focal is used in Hologic Dimensions, W/Rh is used with a lower kVp for thinner CBTs (~55 mm), and then the system switches to W/Ag and uses a higher kVp. Siemens remains mostly within an intermediate kVp range. When a 0.1 focal is used in Siemens, Mo/Rh is used with a lower kVp for thinner CBTs (~49 mm), and then the system switches to W/Rh and uses a higher kVp.

The SBD trend lines show comparatively small steady linear decreases with increasing CBTs. This result likely reflects small geometric adjustments that are inherent to each machine’s design. Hologic Lorad SBD values were unavailable, so these data are not plotted. Despite the lines of the 0.1 focal spot dataset in Siemens that were used for magnification mode, the Siemens 0.3 focal spot dataset was always using smaller SBDs relative to the other machines. 

For CF, the curves diverge more visibly across manufacturers. GE’s lines show modest force increases with thicker breasts, while Hologic Dimensions and Lorad appear to exert higher forces at larger CBT values. Siemens applies intermediate compression levels, although its 0.1 mm focal spot data may deviate from the 0.3 mm focal spot curves at specific thickness intervals.

**Figure 4:**
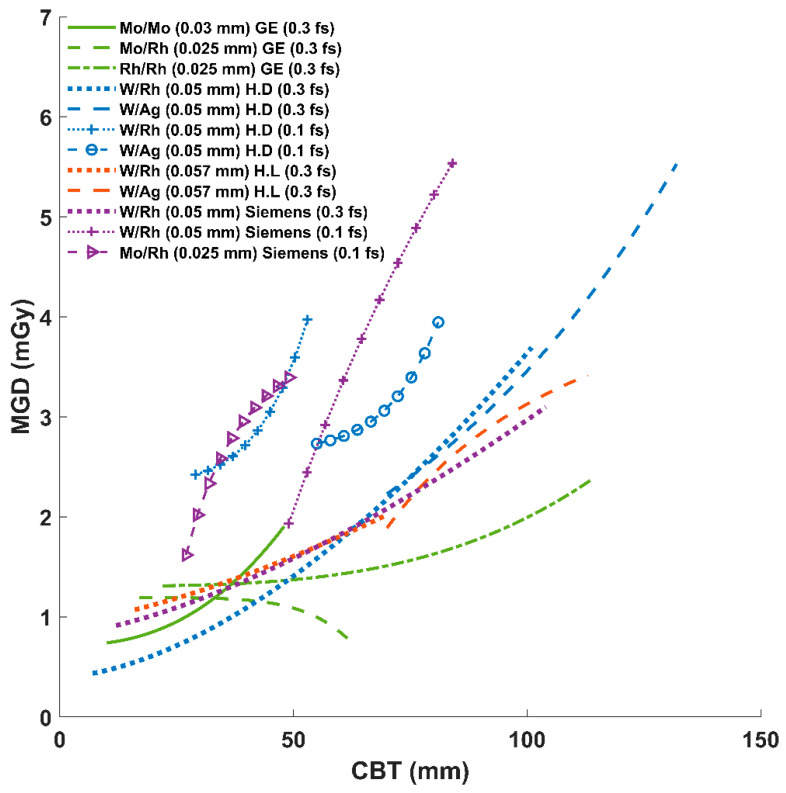
MGD versus compressed breast thickness (CBT) for various AEC-controlled digital mammography systems (GE, Hologic Dimensions (H.D), Hologic Lorad (H.L), and Siemens (Sm.) systems). Each line represents data acquired with different target/filter (and filter thickness) combinations and focal spots (f.s) for some systems. Colors are assigned by manufacturer (GE, green; Hologic Dimensions, blue; Hologic Lorad, orange; Siemens, purple), and line styles differentiate target/filter combinations.

The regression analysis of MGD versus CBT (Figure 4) displays how the MGD varies with the CBT for multiple target/filter combinations and focal spot sizes across four manufacturers’ AEC-controlled digital mammography systems. Overall, the MGD rises as the CBT increases. However, among the GE curves, the MGD increases steadily but remains comparatively moderate up to ~100 mm CBT. Hologic Dimensions and Lorad lines span a wider dose range, particularly at higher CBT values, suggesting that these systems may use higher exposure adjustments to maintain image quality. Siemens 0.3 focal spots have MGD responses that are close to those of Hologic systems, especially Lorad. The dose increase with data of 0.1 focal spots is faster than those acquired with 0.3 focal spots, regardless of the system used.

## 4. Discussion

This study investigated how different AEC strategies by various digital mammography systems impact the delivered MGD by analyzing a large in vivo dataset of 194,608 mammograms. By comparing the dose that is delivered across systems and evaluating exposure parameters (mAs and kVp) alongside the SBD and CF in relation to the CBT, our work provides a comprehensive assessment of dose optimization in clinical settings. Rather than simulations and phantom studies, this study provided results based on a realistic and more complicated setup, with in vivo data acquired in clinical settings. Our analysis confirmed that digital mammography systems exhibit significant inter-system variability in MGD, mostly due to differences in AEC behavior and anode/filter selection.

As can be seen from Table 1 and Figure 3A,B, each manufacturer’s AEC algorithm adapts its exposure parameters (kVp, mAs) differently with increasing CBTs, and the target/filter combination also varies with the CBT. For the GE Senographe Essential, the system transitions from Mo/Mo to Mo/Rh and then Rh/Rh as the CBT increases, following the suggested order in an earlier Monte Carlo study [15]. For the Hologic Dimensions and Lorad systems, they generally begin with W/Rh for thinner breasts and switch to W/Ag for larger CBTs, a strategy that is supported by earlier phantom and simulation studies [16,17]. The Siemens Mammomat Inspiration uses W/Rh for all CBTs unless the magnification mode is activated, in which case it uses 0.1 focal spot and starts with Mo/Rh for thinner CBTs (around 49 mm) before switching to W/Rh. For 0.1 mm focal spot data in the Hologic Dimensions system, it uses W/Rh with thinner CBTs (approximately 55 mm) before switching to W/Ag. Thus, although all systems make their adjustments based on the CBT, their technological implementations vary. 

Our results confirmed that the MGD increases with the CBT across all systems (see Figure 1 and Figure 4), with a general trendline that is aligned with results reported in previous studies [18]. However, the rate and pattern of this increase differ markedly between manufacturers. As can be seen in Figure 1 and Figure 4, the GE Senographe Essential showed a more gradual increase in MGD with CBT, whereas the Hologic (both Dimensions and Lorad models) and Siemens Mammomat Inspiration systems demonstrated steeper dose rises. These findings are in line with Kanaga et al.’s phantom study, which reported that Hologic units tended to deliver higher doses compared to GE when imaging thicker breasts [19]. These variations in AEC settings between manufacturers lead to variations in dose delivery for similar breast thicknesses, potentially impacting the image quality and patient safety. These findings emphasize the need for standardized dose optimization protocols to ensure consistent radiation exposure while maintaining diagnostic image quality across different mammographic systems. 

The Kruskal–Wallis test followed by the pairwise comparisons (Figure 2) revealed significant differences in the majority of pairwise comparisons (26 out of 28), indicating substantial variations in radiation dose optimization across different systems and target/filter combinations. Although a couple of configurations with different systems and target/filter combinations did not differ significantly, this could have occurred due to their physical differences. For instance, Hologic Dimensions with W/Ag(0.05 mm) versus Hologic Lorad with W/Rh(0.057) showed no significant differences in MGD per CBT, which may be due to the effect of their different filter thicknesses (thicker filters result in harder beams and a lower dose). Also, Hologic Lorad with W/Ag(0.057) versus Siemens with W/Rh(0.05) did not differ significantly, which may be because of the differences in system configuration affecting the SBD (Siemens’s SBD is always shorter, which may lead to higher doses). This finding also suggests that comparable dose outcomes might be achieved despite employing target/filer, but this might require more complicated efforts than standardizing technologies among manufacturers. 

Even within systems supplied by the same manufacturer (Hologic) that use identical target/filter materials, a small shift in the delivered dose was observed due to differences in filter thickness. As illustrated in Figure 3A, the mAs used with W/Ag is higher in the Hologic Dimensions system (which uses a 0.05 mm filter thickness) than in the Hologic Dimensions Lorad system (which uses a 0.057 mm filter thickness) at similar CBTs, resulting in a slightly higher dose (Figure 2). This highlights that even small adjustments in technology can influence dose optimization. 

Among the applied technologies, GE uses thinner filters and lower kVp (see Table 1 and Figure 3B), the lowest mAs (Figure 3A), and the highest CF (Figure 3D). This resulted in the overall lowest MGD per CBT (Figure 1), except when Mo/Mo(0.03) is applied (Figure 2). Although Mo/Mo(0.03 mm) uses the lowest kVp in GE (see Figure 3B and Table 1), it resulted in the highest dose per CBT (Figure 2), which is mostly caused due to the lower radiation penetration ability when applied to thicker breasts. This can be noticed in Figure 4, where the MGD increased with MoMo (0.03 mm), particularly when applying it to the larger CBTs in its range (see Figure 4). Thus, for thicker CBTs (≥51 mm; see Figure 1 and Figure 4), adopting technologies that are similar to those used in GE systems (employing Mo/Rh(0.025 mm) and then switching to Rh/Rh(0.025 mm) may help reduce the delivered dose. For thinner CBTs, adapting Hologic Dimensions technology with a W/Rh(0.05 mm) within a configuration allowing for a relatively large SBD (see Figure 3C) might be preferred, as it provided the lowest MGDs in the thinner CBT range (Figure 4). Moreover, the integration of Tungsten-based anodes, as evaluated by a previous phantom study, has led to improved dose efficiency [20], a finding that our clinical data also support. 

It is worth noting that, although GE systems delivered the lowest MGD per CBT, the reduced dose may influence image quality metrics, which is an aspect that was not assessed in this study. Future studies should evaluate whether lower-dose systems maintain adequate diagnostic performance across the full range of breast thicknesses.

The variation in technology can also be noticed with the 0.1 focal spot data. Data from Siemens acquired with a 0.1 mm focal spot generally use low mAs values, showing only a modest increase with the W/Rh configuration but a more pronounced rise with Mo/Rh. In contrast, the Hologic Dimensions system with a 0.1 mm focal spot operates in an intermediate mAs range, although the W/Ag configuration results in higher doses at lower CBTs compared to W/Rh. Overall, 0.1 mm focal spot data typically require somewhat lower mAs at a given thickness than 0.3 mm focal spot data, although this pattern is not consistent across all systems.

Our regression analyses indicated that the relationship between acquisition parameters (mAs and kVp) and the CBT is not uniform across systems (Figure 3A,B). This reinforces the concept that AEC algorithms are tailored to the unique design and calibration of each system. Consistent with earlier studies, our findings emphasize that technological variations significantly influence radiation dose management, underscoring the necessity for system-specific exposure protocols.

The CF was found to be varying among modalities (Figure 3D). These CF trends likely result from both mechanical design and technologist techniques [21]. Despite regular technical training, operator-dependent variability may explain some of the observed CF differences. Future work with a collected log of technologist identifiers can be conducted to quantify and adjust for this operator-dependent effect.

Despite the strengths of our large, in vivo dataset, our study has some limitations. We only evaluated a limited number of systems. Although we analyzed four widely used systems, additional manufacturers and models may exhibit different AEC behaviors. Expanding the vendor pool in future studies would improve the generalizability of our findings. However, the results of the four systems in this study were enough to show the existence of optimization variability across clinical systems. Another limitation of this study is that the variation in breast composition and imaging protocols across centers may affect the generalizability of our results. Breast density was not incorporated, because it is not directly reported by the mammography systems and would require unavailable software or labor-intensive manual scoring by radiologists, resources that are beyond the scope of this large retrospective dataset. Since there is a possibility for breast density to influence the AEC behavior, future works clarifying its effect would be of interest. Moreover, while the image quality is an important criterion in dose optimization, it was not assessed alongside the MGD in our study. Future work investigating image quality measurements as well would be useful to ensure that dose reductions do not compromise diagnostic performance. Thus, it would be useful in future studies to include a broader range of systems, incorporate prospective designs, and examine the interplay between breast density, image quality, and radiation dose. Also, developing guidelines to help operators determine the appropriate CF would be valuable, given the observed variability in CF strategies (Figure 3D).

The variation in AEC optimization that we observed across systems has critical clinical consequences. While the higher radiation exposure at increased CBTs may improve lesion contrast and sensitivity (potentially enhancing cancer detection rates in thick or dense breasts), it also increases the MGD. Over multiple screening rounds, this can incrementally raise the lifetime risk of radiation-induced malignancies, especially in high-risk groups. Conversely, systems that prioritize dose reduction may lower radiation-associated risks at the expense of degrading the image quality, which can lower the detection sensitivity and drive higher recall or biopsy rates due to suboptimal lesion conspicuity. 

Recognizing the inter-system variability is crucial for optimizing patient care. Clinicians should calibrate and monitor each unit individually rather than applying a one-size-fits-all protocol. Establishing system-specific diagnostic reference levels and conducting regular dose audits can help ensure that patients receive the lowest reasonably achievable dose without compromising the image quality. Additionally, adopting standardized, optimized AEC behaviors and unified benchmarks across platforms for both dose and image quality would facilitate the establishment of harmonized diagnostic reference levels and quality assurance in multicenter screening programs. These are critical to safeguarding patient safety, maintaining a high diagnostic accuracy, and optimizing the overall screening effectiveness. 

In summary, our analysis highlights the complexity of dose optimization in digital mammography, influenced by the AEC algorithm design, target/filter selection, and inherent system geometry. The findings underscore the need for ongoing efforts to standardize AEC performance. Addressing system-specific differences will improve both the safety and effectiveness of breast cancer screening.

## 5. Conclusions

The delivered MGD in digital mammography is significantly affected by the machine technology and the specific acquisition parameters, including the target/filter combinations. Clinicians must be aware of these inter-system variations to optimize imaging protocols on a per-device basis to minimize patients’ radiation exposure, interpret differences in patient dose, and enhance quality control measures. Moreover, standardizing dose optimization strategies across different manufacturers could lead to more consistent diagnostic outcomes and enhanced patient safety. These insights highlight the need for tailored clinical guidelines and quality assurance procedures that consider system-specific AEC behaviors, ultimately driving improvements in digital mammography practice.

## Figures and Tables

**Table 1:** The number of images per machine and their acquisition parameters.

Manufacturer	Model	Number of Images	Target/Filter (Filter Thickness)	CBT [mm]	CF [N]	kVp	mAs	SBD [cm]	MGD [mGy]
				Mean ± SD (Range)					
GE	Senographe Essential *	168,000	Mo/Mo (0.03 mm) [0.64%]	31.8 ± 7.5 (10–48)	138 ± 42 (30–200)	26 ± 0.3 (25–27)	51 ± 18 (5–113)	63 ± 1 (61–65)	1.2 ± 0.4 (0.15–2.3)
			Mo/Rh (0.025 mm) [15.52%]	43 ± 7 (17–63)	141 ± 37 (30–270)	27 ± 0.5 (26–28)	56 ± 14 (6–230)	62 ± 1 (60–64)	1.1 ± 0.3 (0.16–3.7)
			Rh/Rh (0.025 mm) [83.84%]	61 ± 10 (22–114)	138 ± 37 (20–270)	29 ± 0.6 (27–31)	69 ± 19 (2–335)	60 ± 1 (55–64)	1.5 ± 0.3 (0.12–7.8)
			All combinations	58 ± 12 (10–114)	138 ± 37 (20–270)	29 ± 1.1 (25–31)	67 ± 19 (2–335)	60 ± 1 (55–65)	1.4 ± 0.4 (0.12–7.8)
Hologic	Selenia Dimensions	2660	W/Rh (0.05 mm) [f.s: 0.3; 86.6%]	55 ± 11 (7–101)	115 ± 46 (18–312)	30 ± 2 (25–39)	140 ± 53 (31–462)	62 ± 1 (57–67)	1.6 ± 0.7 (0.4–6.3)
			W/Ag (0.05 mm) [f.s: 0.3; 11.4%]	77 ± 8 (70–132)	111 ± 48 (25.4–301)	31 ± 2 (30–39)	190 ± 59 (57–477)	60 ± 1 (54–61)	2.6 ± 0.9 (0.7–6.9)
			W/Rh (0.05 mm) [f.s: 0.1; 1.1%]	41 ± 6 (29–53)	92 ± 33 (32–150)	29 ± 1 (26–31)	89 ± 16 (67–119)	63 ± 1 (62–64)	2.9 ± 0.6 (2–4.2)
			W/Ag (0.05 mm) [f.s: 0.1; 0.9%]	65 ± 7 (55–81)	74 ± 38 (21–158)	32 ± 1 (30–35)	76 ± 21 (54–134)	61 ± 0.7 (59–62)	3 ± 1 (1.6–5.0)
			All combinations	57 ± 13 (7–132)	114 ± 46 (18–312)	30 ± 2 (25–39)	145 ± 56 (31–477)	62 ± 1 (54–67)	1.8 ± 0.8 (0.4–6.9)
Hologic	Lorad Selenia *	23,168	W/Rh (0.057 mm) [80.2%]	53 ± 10 (16–70)	123 ± 55 (44–325)	29 ± 2 (25–32)	128 ± 39 (15–378)	NA	1.7 ± 0.5 (0.2–5.9)
			W/Ag (0.057 mm) [19.8%]	77 ± 6 (70–113)	111 ± 35 (44–320)	29 ± 1 (28–37)	169 ± 41 (14–404)	NA	2.3 ± 0.6 (0.2–6.4)
			All combinations	58 ± 13 (16–113)	121 ± 52 (44–325)	29 ± 2 (25–39)	136 ± 42 (14–404)	NA	1.8 ± 0.6 (0.2–6.4)
Siemens	Mammomat Inspiration	780	W/Rh (0.05 mm) [f.s: 0.3; 66%]	58 ± 15 (12–104)	74 ± 37 (23–196)	29 ± 1 (25–32)	166 ± 82 (47–488)	58 ± 1.5 (53–62)	1.8 ± 0.8 (0.6–5.0)
			W/Rh (0.05 mm) [f.s: 0.1; 22.5%]	60 ± 8 (49–84)	50 ± 20 (27–145)	30 ± 1 (29–32)	96 ± 37 (46–200)	33 ± 0.8 (31–34)	3.3 ± 1.2 (1.7–6.8)
			Mo/Rh (0.025 mm) [f.s: 0.1; 11.5%]	40 ± 6 (27–49)	56 ± 22 (28–124)	27 ± 1 (26–28)	43 ± 14 (21–93)	35 ± 0.6 (34–36)	3.0 ± 0.9 (1.6–6.2)
			All combinations	56 ± 14 (12–104)	67 ± 34 (23–196)	29 ± 1 (25–32)	136 ± 82 (21–488)	50 ± 11 (31–62)	2.3 ± 1.1 (0.6–6.8)

CBT, compressed breast thickness; kVp, kilovoltage peak; mAs, milliampere-seconds; CF, compression force; SBD, source-to-breast distance; MGD, mean glandular dose; SD, standard deviation; f.s, focal spot in mm; Mo, Molybdenum; W, Tungsten; Rh, Rhodium; Ag, Silver; NA, not available. * All data from these systems were acquired with a 0.3 mm focal spot.

## Data Availability

The data presented in this study are available on request from the corresponding author.
